# Effects of dietary supplementation with *Pediococcus acidilactici* ZPA017 on reproductive performance, fecal microbial flora and serum indices in sows during late gestation and lactation

**DOI:** 10.5713/ajas.18.0764

**Published:** 2019-05-27

**Authors:** Hui Liu, Sixin Wang, Dongyan Zhang, Jing Wang, Wei Zhang, Yamin Wang, Haifeng Ji

**Affiliations:** 1 Institute of Animal Husbandry and Veterinary Medicine, Beijing Academy of Agriculture and Forestry Sciences, Beijing 100097, China

**Keywords:** Fecal Microbial Flora, *Pediococcus acidilactici*, Reproductive Performance, Serum Indices, Sows

## Abstract

**Objective:**

This study was conducted to determine the effects of dietary supplementation with *Pediococcus acidilactici* (*P. acidilactici*) ZPA017 as a probiotic on reproductive performance, fecal microbial flora and serum indices in sows during late gestation and lactation.

**Methods:**

A total of 94 sows (Large White×Yorkshire, average 4.50 parities) were randomly allotted to two dietary treatments: control diet and the diet supplemented with *P. acidilactici* ZPA017 (2.40×10^9^ colony-forming unit/kg of diets). The study started at d 90 of gestation and conducted until d 28 of lactation.

**Results:**

Compared to sows fed the control diet, supplementation of *P. acidilactici* ZPA017 increased the number of weaning piglets, weaning weight of litter and piglets, survival rate of piglets at weaning (p<0.05), and decreased diarrhea rate of piglets in lactation (p<0.05). Dietary *P. acidilactici* ZPA017 increased fecal *Lactobacillus* populations (p = 0.030) and reduced fecal *Escherichia coli* and *Staphylococcus aureus* populations (p<0.05) of sows at weaning. Moreover, the supplementation of *P. acidilactici* ZPA017 increased serum concentrations of immunoglobulin G, immunoglobulin A and total protein (p<0.05), while decreased serum haptoglobin concentration and alanine aminotransferase activity (p<0.05) of sows at weaning.

**Conclusion:**

Administration of *P. acidilactici* ZPA017 in diets during late gestation and lactation had positive effects on the reproductive performance, intestinal microflora balance and immunity of sows.

## INTRODUCTION

Although antibiotics have been used in diets for pregnant sows to improve the viability of their progeny in the past several decades [1–3], antibiotics-resistant microbes are a growing issue that has been exacerbated by the overuse and misuse of antibiotics. Therefore, it is urgent to find alternatives for antibiotics especially after the ban on the use of antibiotics as a growth promoter in Europe since 2006. Probiotics have received increasing interest as an alternative to maintain animal health and performance.

Probiotics are defined as live microorganisms that, when administered in adequate amounts, confer a health benefit on the host [4]. Several studies have evaluated the effects of probiotics on the reproductive performance of sows. Böhmer et al [5] reported that dietary *Enterococcus faecium* DSM 7134 in sows from d 90 of pregnancy to d 28 of lactation showed an improvement of lactation feed intake, litter size and weight performance of sows. Alexopoulos et al [6] investigated the effects of a dietary probiotic (containing *Bacillus licheniformis* and *Bacillus subtilis* spores) in sows and reported that the probiotic improved the reproductive performance of sows, with reduced piglet diarrhea, reduced pre-weaning mortality, and increased weaning weights. However, to date, little research has been performed to determine the effects of *Pediococcus acidilactici* (*P. acidilactici*) on sows.

*Pediococcus acidilactici* is a gram-positive, coccus-shaped, lactic acid- fermenting bacterium that belongs to the *Lactobacillaceae* family. *P. acidilactici* has been used as a probiotic for pigs, poultry, aquaculture and human dietary supplements [7,8]. *P. acidilactici* ZPA017 was isolated from the feces of a healthy pig in our laboratory. The strain was identified through standard morphological, biochemical and physiological tests by 16s rRNA gene sequence analysis at the China General Microbiological Culture Collection Center (Beijing, China). Therefore, the aim of this study was to investigate the effects of *P. acidilactici* ZPA017 on the reproductive performance, fecal microbial flora and serum indices in sows during late gestation and lactation.

## MATERIALS AND METHODS

### Ethics statement

The study was carried out in accordance with the recommendations of guidelines set by the Animal Care and Use Committee (permit number: SYXK-2017-0005) of the Institute of Animal Husbandry and Veterinary Medicine, Beijing Academy of Agriculture and Forestry Sciences (IAHVM-BAAFS), Beijing, China. The protocols were approved by the Animal Care and Use Committee of IAHVM-BAAFS.

### Animals, diets, and housing

A total of 94 sows (Large White×Yorkshire, average 4.50 parities) were randomly allotted to 2 dietary treatments including control diet with no probiotic and probiotic diet supplemented with *P. acidilactici* ZPA017 (2.40×10^9^ colony-forming unit/kg of diets) based on parity and body weight. All diets were formulated to meet or exceed all nutrient requirement of NRC [9]. The ingredient and chemical composition of the basal diet for gestating and lactating sows are shown in Table 1. In this study, *P. acidilactici* ZPA017 freeze-dried powder was incorporated in the probiotic diet via corn substitution. The trial started at d 90 of gestation and conducted until d 28 of lactation. From d 90 to d 107 of gestation, all sows were housed in individual gestation stalls (2.2×0.7 m^2^) and fed gestation diets. Then, sows were moved to farrowing crates (2.5×1.8 m^2^) and were fed lactation diets from d 108 of gestation until weaning. The farrowing room temperature was maintained at 18°C to 25°C with 60% relative humidity. From d 90 of gestation until the day of farrowing, the sows were fed 3.0 kg/d of diet. On the day of farrowing, sows were fasted. The sows were fed 2.0 kg diet on d 1 of lactation. Thereafter, the amount of feed allowance increased gradually (1.0 kg each day) for the first week. Sows were then fed *ad libitum* until weaning at d 28 of lactation. The diets were supplied twice a day (07:00 and 15:00) and sows had *ad libitum* access to water throughout the experimental period.

### Performance measurement

Individual sows were weighed at d 108 of gestation and d 28 of lactation to determine weight loss. The feed intake during lactation was recorded for each sow to calculate average daily feed intake. Within 12 h after birth, the number of piglets born, born alive and dead were recorded. Piglets were individually weighed at birth and weaning (d 28 of lactation) to calculate average daily gain. At weaning, the number of weaned piglets was recorded. The diarrhea incidence in piglets within litters was evaluated according to a previous study [10] as follows: Diarrhea incidence (%) = (Total number of pigs with diarrhea)/(total number of pigs×experimental days) ×100. Fresh excreta were graded using the following scale: 0 = solid, 1 = semisolid, 2 = semiliquid, and 3 = liquid. The occurrence of diarrhea was defined as production of grade 2 or 3 feces for 2 continuous days.

### Fecal samples collection and microbiological determination

To determine the microbiological status of sows, fresh fecal samples were collected from the sows (10 randomly selected sows per treatment with the similarly parity) on d 28 of lactation. The microbiological assay of fresh fecal samples was carried out as previously described by Choi et al [11]. Fecal samples were subjected to a series of 10-fold dilutions in sterile physiological saline and incubated in duplicate on selective medium. The colony counts were then enumerated after culturing in Petri plates with the corresponding culture media: MacConkey agar for *Escherichia coli* (*E. coli*), Baird-Parker agar for *Staphylococcus aureus* (*S. aureus*), and de Mann, Rogosa and Sharpe (MRS) agar for *Lactobacillus*. The plates were incubated at 37°C for 48 h. Bacterial counts were log transformed before statistical analysis.

### Blood collection and analysis

Blood samples (5 mL) were obtained using vacuum blood collection tubes from an ear vein of 10 randomly selected sows per treatment with a similarly parity on the final day of the trial. After centrifugation (3,000×*g* for 15 min at 4°C), serum was collected and stored at −20°C until analysis. Enzyme-linked immunosorbent assays were used to determine the concentrations of immunoglobulins (IgG, IgM, and IgA; Bethyl Laboratories, Inc., Montgomery, TX, USA) and haptoglobin (alpha diagnostic international, San Antonio, TX, USA). Serum concentrations of albumin, globulin, total protein, glucose, blood urea nitrogen and the serum activities of alanine aminotransferase and aspartate aminotransferase were determined using automatic biochemical analyzer (Model 7020; Hitachi, Tokyo, Japan) with corresponding kits (Nanjing Jiancheng Bioengineering Institute, Nanjing, China).

### Statistical analysis

All experimental data were analyzed using the general linear model procedure of SAS version 9.2 (SAS Institute, Inc., Cary, NC, USA) as a randomized complete block design according to their body weight. The individual sow or litter of piglets was used as experimental unit. The analysis of piglets weaning weight used birth weight as covariates. Lactation length was used as a covariate for piglets survivability, sows and piglets weaning weight, sow feed intake and piglets average daily gain. Duncan’s multiple range test, and a probability level of p<0.05 was regarded as statistically significant.

## RESULTS

Dietary *P. acidilactici* ZPA017 increased the number of piglets at weaning, survival rate at weaning, weight of piglets at weaning and litter weight at weaning (p<0.05), and decreased diarrhea incidence of piglets (p<0.05) compared with sows fed control diet (Table 2).

Dietary *P. acidilactici* ZPA017 increased fecal *Lactobacillus* populations (p = 0.030) and reduced fecal *E.coli* and *S. aureus* populations (p<0.05) compared with sows fed control diet (Table 3).

Compared with sows fed control diet, supplementation of *P. acidilactici* ZPA017 in diets increased serum IgG, IgA, and total protein concentrations (p<0.05), while decreased serum haptoglobin concentration and alanine aminotransferase activity (p<0.05) of sows at weaning (Table 4).

## DISCUSSION

Dietary supplementation of probiotics can increase the production and activities of digestive enzymes in the gut, improve gut health and nutrient digestibility, and therefore benefit nutrient utilization and growth performance of pigs [12–14]. Previous studies have shown that supplementation of probiotics in diets could improve the reproductive performance and health status of sow and litter [15–17]. In the present study, dietary supplementation with *P. acidilactici* ZPA017 increased weight of piglets at weaning and litter weight at weaning. This result is similar to the findings of Kim et al [18], who reported that higher litter weight was observed when sows were fed yeast culture during gestation and lactation. The present study also indicated that in comparison to sows fed diets without probiotics, the number of piglets at weaning and survival rate increased. Similarly, to our results, Taras et al [19] reported that the rate of survival piglets at weaning were improved in sows fed *Enterococcus faecium* NCIMB 10415. Additionally, in the present study, *P. acidilactici* ZPA017 supplementation to sows during late gestation and lactation reduced the incidence of diarrhea in their piglets, which was consistent with the result of Alexopoulos et al [6] who reported a lower diarrhea score in piglets from sows administered with *Bacillus licheniformis* and *Bacillus subtilis*. These results in current study suggested that the administration of *P. acidilactici* ZPA017 could improve the health and reproductive performance of sows. One possible explanation could be that the improved microbial balance in the gut is optimized and a better utilization of nutrients is taking place, improving the health status of sows.

Lactic acid bacteria could produce short chin fatty acids and lactic acid, which may help to improve the intestinal microbial balance by reducing the pH of the intestinal content and inhibiting the growth and multiplications of opportunistic enteropathogens such as *Salmonella* spp. or strains of *E. coli* [20–22]. In the current study, *P. acidilactici* ZPA017 supplementation to diets for sows reduced the fecal *E. coli* and *S. aureus* concentration and increased the fecal *Lactobacillus* concentration. This indicated that the strain *P. acidilactici* ZPA017 could regulate the balance of intestinal microflora and establish a healthier gut, which had beneficial effects in improving the health and reproductive performance of sows, as supported by the results in this study. However, Scharek et al [23] demonstrated no differences in fecal bacterial populations of sows administered with *Enterococcus faecium* SF68 compared with those of the control group. Pollmann [24] reported that the fecal *Lactobacillus* and *E. coli* counts were not affected when feeding sows with a diet contained *Bacillus*. The inconsistent results with fecal bacterial populations between the current study and previous reports may be owing to the differences in strains of probiotics, concentrations of probiotics in diets, nutrition level and diet sources, housing environment and the age of sows, but further investigations are needed to verify.

Probiotics have the capacity to stimulate the immune system, which is very relevant in pig production to avoid losses related to health reasons (e.g. decreased milk production) of sows and piglets during the peripartal period [25–27]. In pigs, the immunoglobulin concentration is an important immune parameter [28,29]. Results of the current study showed that diets supplemented with *P. acidilactici* ZPA017 increased serum IgG and IgA concentrations in sows, demonstrating that *P. acidilactici* ZPA017 had beneficial effect on immune function so as to improve health status and performance of sows. This result agreed with a previous study showing that *Lactobacillus johnsonii* XS4 supplementation increased the serum IgG concentration in sows [30]. However, the mechanism for enhancing immune function by *P. acidilactici* was not clear, the reason might be that the probiotic could regulate intestinal microflora and stimulate intestinal tissue to produce immunoglobulin [31,32]. In addition, the enhancement of immunoglobulin in serum could be beneficial to piglets because immunoglobulin can be translocated from blood to colostrum [33,34], which provides humoral immune protection for the new-born piglets and possibly protects them against potential diseases [35]. Although the sows’ colostrum and milk immunoglobulin levels were not observed in this study, the decreased diarrhea incidence of piglets in sows fed *P. acidilactici* ZPA017 might be related to the improved serum immunoglobulin concentrations in sows.

Sows with dietary *P. acidilactici* ZPA017 supplementation had lower serum haptoglobin concentration than sows fed control diets. Haptoglobin is one type of acute phase proteins and has been identified as a sensitive indicator of health status of pigs [36]. Its concentration has been found to increase in the serum of pigs suffering from inflammation, stress and infection or tissue injury [37,38]. The lower haptoglobin concentration suggested that *P. acidilactici* ZPA017 had a beneficial effect helping sows to sustain a good health status. In terms of serum biochemical indicators, total protein is an indicator of adequacy of quantity and quality of diets as well as efficiency of utilization of the dietary protein by the pig [39,40], the increased total protein concentration in sows with dietary supplementation with *P. acidilactici* ZPA017 suggested that the crude protein digestibility was improved and more protein was available for utilization [41]. Alanine aminotransferase is principally found in the liver and is a descriptor of hepatic disease and liver cytolysis in pigs [42]. The lower level of alanine aminotransferase activity in sows supplemented with *P. acidilactici* ZPA017 compared with sows fed control diets indicated that *P. acidilactici* ZPA017 supplementation could alleviate the liver damage and improve the normal function of liver [43]. Similar trends were also observed by Wang et al [30], who showed that *Lactobacillus johnsonii* XS4 supplementation increased serum total protein and decreased alanine aminotransferase levels in sows.

## CONCLUSION

In conclusion, *P. acidilactici* ZPA017 supplementation to sows during late gestation and lactation had positive effects on reproductive performance, intestinal microflora balance and immunity. The present study showed that *P. acidilactici* ZPA017 could be an alternative to antibiotics for use as a feed additive in diets of sows. Further studies are needed to investigate the proper dosage to be administered and the possible mechanism of probiotic actions of *P. acidilactici* ZPA017.

## Figures and Tables

**Table 1:** Ingredients and chemical composition of the control diets (as-fed basis)

Items	Gestation	Lactation
Ingredient composition (g/kg)		
Corn	664.3	650.5
Wheat bran	120	50
Soybean meal	170	220
Fish meal	-	20
Soy oil	10	20
Limestone	11	12
Dicalcium phosphate	10	12
Salt	4	4
L-lysine·HCl (98%)	0.7	1.5
Vitamin and mineral premix for pregnant sows1)	10	-
Vitamin and mineral premix for nursing sows2)	-	10
Chemical composition (%)		
Digestible energy (MJ/kg)3)	13.30	13.80
Crude protein4)	14.50	18.05
Calcium4)	0.78	0.92
Total phosphorus4)	0.54	0.62
Lysine3)	0.72	1.05
Methionine+cysteine3)	0.38	0.57

1) Provided the following quantities per kilogram of complete diet: vitamin A, 10,000 IU; vitamin D_3_, 2,000 IU; vitamin E, 30 mg; vitamin K_3_, 1.5 mg; vitamin B_1_, 2.5 mg; vitamin B_2_, 5.5 mg; vitamin B_6_, 2.5 mg; vitamin B_12_, 0.02 mg; biotin, 0.2 mg; niacin, 25 mg; pantothenic acid, 13 mg; choline chloride, 200 mg; 10 mg of Cu as CuSO_4_·5H_2_O; 80 mg of Fe as FeSO_4_·H_2_O; 25 mg of Mn as MnSO_4_·H_2_O; 100 mg of Zn as ZnSO_4_·H_2_O; 0.15 mg of I as K; 0.3 mg of Se as Na_2_SeO_3_.

2) Provided the following quantities per kilogram of complete diet: vitamin A, 7,000 IU; vitamin D_3_, 1,500 IU; vitamin E, 80 mg; vitamin K_3_, 1.5 mg; vitamin B_1_ 1.8 mg; vitamin B_2_, 5.5 mg; vitamin B_6_, 4 mg; vitamin B_12_, 0.03 mg; biotin, 0.2 mg; niacin, 25 mg; pantothenic acid, 13 mg; choline chloride, 200 mg; 20 mg of Cu as CuSO_4_·5H_2_O; 100 mg of Fe as FeSO_4_·H_2_O; 25 mg of Mn as MnSO_4_·H_2_O; 100 mg of Zn as ZnSO_4_·H_2_O; 0.5 mg of I as KI; 0.3 mg of Se as Na_2_SeO_3_.

3) Calculated value.

4) Analyzed value.

**Table 2:** Effects of dietary *Pediococcus acidilactici* ZPA017 supplementation on reproductive performance of sows during late gestation and lactation

Items	Control1)	Probiotic1)	SEM	p-value
Sows				
Number of sows	47	47		
Average parity	4.89	4.51	0.181	0.954
Sow body weight on day 108 of gestation (kg)	281.3	278.9	3.769	0.541
Sow body weight at weaning (kg)	248.2	241.4	3.333	0.237
Body weight change (kg)	33.1	37.5	1.077	0.575
Lactation average daily feed intake of sows (kg/d)	4.90	5.00	0.222	0.304
Total born/litter	11.57	11.63	0.362	0.930
Born alive/litter	11.04	11.26	0.335	0.761
Live-born rate (%)	96.52	96.81	0.848	0.441
Piglets				
Litter weight of live-born piglets at birth (kg)	15.43	16.51	0.532	0.323
Weight of live-born piglets at birth (kg)	1.42	1.48	0.044	0.433
Litter weight at weaning (kg)	60.45	69.33	2.441	0.037
Weight of piglets at weaning (kg)	6.19	6.65	0.115	0.049
Number of piglets at weaning	9.65	10.37	0.293	0.031
Survival rate at weaning (%)	88.68	92.42	1.480	0.023
Average daily gain (g/d)	170.33	184.91	4.532	0.110
Diarrhea rate of piglets (%)2)	8.30	3.38	0.064	0.023

SEM, standard error of means; CFU, colony-forming unit.

1) Control, control diet; Probiotic, control diet supplemented with *Pediococcus acidilactici* ZPA017 (2.40×10^9^ CFU/kg of diets).

2) Diarrhea incidence (%) = (Total number of pigs with diarrhea)/(total number of pigs×experimental days)×100.

**Table 3:** Effects of dietary *Pediococcus acidilactici* ZPA017 supplementation on fecal microbial populations of sows at weaning (log_10_ CFU/g)

Items	Control1)	Probiotic1)	SEM	p-value
*Escherichia coli*	7.63	6.43	0.324	0.038
*Staphylococcus aureus*	6.26	5.98	0.272	0.047
*Lactobacillus*	8.51	9.11	0.151	0.030

SEM, standard error of means; CFU, colony-forming unit.

1) Control, control diet; Probiotic, control diet supplemented with Pediococcus acidilactici ZPA017 (2.40×10^9^ CFU/kg of diets).

**Table 4:** Effects of dietary *Pediococcus acidilactici* ZPA017 supplementation on serum indices of sows at weaning

Items	Control1)	Probiotic1)	SEM	p-value
Immunoglobulin G (mg/mL)	4.64	5.61	0.320	0.032
Immunoglobulin A (mg/mL)	0.96	1.24	0.167	0.044
Immunoglobulin M (mg/mL)	1.27	1.47	0.150	0.133
Haptoglobin (mg/mL)	0.74	0.62	0.142	0.032
Albumin (g/L)	31.27	30.19	1.118	0.333
Globulin (g/L)	29.01	35.08	1.244	0.450
Total protein (g/L)	60.28	65.27	1.983	0.047
Glucose (mmol/L)	4.83	4.80	0.444	0.311
Blood urea nitrogen (mmol/L)	4.30	4.08	0.121	0.232
Alanine aminotransferase (U/L)	34.00	28.98	3.580	0.044
Aspartate aminotransferase (U/L)	39.23	37.19	4.333	0.600

SEM, standard error of means; CFU, colony-forming unit.

1) Control, control diet; Probiotic, control diet supplemented with *Pediococcus acidilactici* ZPA017 (2.40×10^9^ CFU/kg of diets).
